# Arterial stiffness shown by the cardio-ankle vascular index is an important contributor to optic nerve head microcirculation

**DOI:** 10.1007/s00417-016-3521-9

**Published:** 2016-10-14

**Authors:** Tomoaki Shiba, Mao Takahashi, Tadashi Matsumoto, Kohji Shirai, Yuichi Hori

**Affiliations:** 1 Department of Ophthalmology, School of Medicine Toho University, Tokyo, Japan; 2 Cardiovascular Center, Toho University Sakura Medical Center, 564-1 Shimoshizu, Sakura, Chiba 285-8741 Japan; 3 Internal Medicine, Toho University Sakura Medical Center, Sakura, Chiba Japan

**Keywords:** Cardio-ankle vascular index, Ocular circulation, Laser speckle flowgraphy, Pulse waveform analysis, Arteriosclerosis

## Abstract

**Propose:**

The purpose of this study was to determine whether there were significant correlations between the pulse waveform in the capillary area of the optic nerve head (ONH) microcirculation shown by laser speckle flowgraphy (LSFG) and parameters of the systemic condition, especially the cardio-ankle vascular index (CAVI).

**Method:**

We studied 130 men (ages 60.5 ± 10.9 years) who visited the Vascular Function Section of the Department of Cardiovascular Center of Toho University Sakura Medical Center. We evaluated the skew and blowout time (BOT) — which are parameters of pulse waveform analyses — using LSFG in the capillary area of the ONH for each patient. The CAVI, the E/e’ ratio as the measure of diastolic left ventricular function, and the mean intima-media thickness (IMT) were evaluated as systemic parameters. We performed a Pearson’s correlation analysis and a multiple regression analysis to determine independent factors for skew and BOT.

**Results:**

Heart rate, spherical refraction, and the CAVI (standard regression = 0.18, *t* = 2.61, *p* = 0.01) were revealed as factors contributing independently to the skew by multiple regression analysis. Heart rate, the CAVI (standard regression = −0.27, *t* = −3.92, *p* = 0.0002), the urinary albumin concentration, the mean IMT, spherical refraction, body mass index and pulse pressure were revealed as factors contributing independently to the BOT by multiple regression analysis.

**Conclusion:**

The CAVI was demonstrated to be an independent factor contributing to both skew and BOT in the capillary area of the ONH. Our findings clarified that large arterial function shown by the CAVI contributes to smooth hemodynamics of microcirculation, shown by LSFG.

## Introduction

Systemic arteriosclerosis is an important contributing factor to cardiovascular disease and cerebrovascular disease, accounting for much of the mortality due to these diseases [1]. An arterial stiffness parameter, the cardio-ankle vascular index (CAVI), was developed as a marker related to arteriosclerosis including that of the aorta, femoral artery, and tibial artery [2]. The CAVI is theoretically independent of blood pressure, because the CAVI is measured from the stiffness parameter, β, which was proposed by Hayashi et al. [3] The CAVI is based on the variance of the arterial pressure that is required to change the vascular diameter.

There are also several reports of relationships between systemic arteriosclerosis and ocular diseases, such as retinal vein occlusion- and age-related macular degeneration [4–9]. It is thus clear that important risk factors for cardiovascular disease, cerebrovascular disease and retinal disease overlap. This concept is supported by the finding that the ocular microcirculation provides a window for detecting changes in microvasculature related to the development of cardiovascular diseases [10].

Laser speckle flowgraphy (LSFG), a noninvasive quantitative method of determining ocular blood flow [11, 12], is based on the changes in the speckle pattern of laser light reflected from the fundus of the eye [13, 14]. LSFG is dependent on the movement of erythrocytes in the retina, the choroid and the optic nerve head (ONH), and the mean blur rate (MBR) is an indicator of ocular blood flow [15, 16]. In 2008, the LSFG-NAVI™ (Softcare Co., Fukuoka, Japan) was approved as a medical apparatus by the Pharmaceuticals and Medical Devices Agency in Japan. Changes in the MBR have various pulse wave patterns that are synchronized with the cardiac cycle.

We have focused on the relationship between the pulse waveform and patients’ systemic conditions, and we reported that the pulse waveform in the ONH was significantly correlated with the CAVI, the left ventricular function obtained from echocardiography, and the carotid intima media thickness (IMT) as shown by ultrasonographic imaging, in healthy subjects [17, 18]. Other researchers clarified that the skew and blowout time (BOT) in the capillary area of the ONH (which are factors derived from pulse waveform analyses) are important predictive factors for normal tension glaucoma and silent brain infarction [19, 20]. In light of all of these findings, we hypothesized that systemic conditions are affected by the skew and BOT in the capillary area of the ONH.

The purpose of this study was thus to determine whether there are significant correlations between the skew and BOT in the capillary area of the ONH shown by LSFG and parameters of the systemic condition, such as the CAVI, mean IMT and left ventricular diastolic function in patients with a variety of disorders.

## Materials and methods

We analyzed the cases of 154 consecutive men who visited the Vascular Function Section of the Department of Cardiovascular Center of Toho University Sakura Medical Center in the years from April 1, 2007 to December 1, 2012. Patients who had glaucoma, moderate degree of cataract, vitreous and retinal diseases, and those who underwent intraocular surgery were excluded. The patients with the following conditions were excluded because these conditions can affect the CAVI and LSFG measurement: chronic atrial fibrillation had decreased the patient’s left ventricular function (ejection fraction <50 %), and peripheral arterial disease defined as an ankle-brachial index <0.9. In the end, 130 patients whose mean ± standard division (SD) age was 60.5 ± 10.9 years with the range 29 to 83 years met the study criteria. The Institutional Review Board of Toho University Sakura Medical Center approved the protocol of this study, and we began the research after all patients received information on the purpose and possible side effects of the research protocol and signed an informed consent. The procedures used conformed to the tenets of the Declaration of Helsinki.

Echocardiography, CAVI measurement, and LSFG were performed after the patient rested for 10 min in a quiet, air-conditioned room with the temperature maintained at 24 ° C. All patients abstained from smoking, alcohol and caffeine for at least 24 h prior to the measurements. All evaluations were made between 15:00 and 17:00 h before a meal.

## Measurements of echocardiography

Left ventricular diastolic function was assessed according to the recent consensus guidelines [21, 22] on diastolic function evaluation, measuring the mitral inflow velocities (E-wave) using pulse wave Doppler in the apical four-chamber view. The pulse wave tissue-Doppler velocities were acquired at end-expiration, in the apical four-chamber view, with the sample positioned at the lateral mitral annulus, measuring early diastolic (e’ velocity) and calculating the E/e’ ratio. The E/e’ ratio has been reported to be the single best predictor of the left ventricle diastolic filling pressure [23]. Echocardiography was performed using a commercially available instrument (Vivid 7, GE Healthcare, Tokyo).

## Measurements of mean IMT

High-resolution ultrasonographic imaging of the carotid artery using the B-scan mode was performed using the EUB-8500 ultrasound system (Hitachi, Tokyo) with the probe frequency set to 7.5 MHz. The imaging was performed with the patient in the supine position with his head turned slightly away from the sonographer. The procedure involved scanning the near and far walls of the carotid artery every 1 cm proximal to the carotid bulb in the longitudinal view. The mean IMT was defined as the average of the maximal IMT 1 cm proximal and 1 cm distal to the carotid bulb [24, 25]. The mean IMT of the right side of the carotid artery was used for the data analyses.

## Measurement of the CAVI, mean arterial blood pressure, and pulse pressure

The measurements of the CAVI, blood pressure, and heart rate were performed using the program embedded in the VaSera VS-1000, a vascular screening system (Fukuda Denshi, Tokyo). A detailed explanation of the methods used to measure the CAVI has been reported [2]. Briefly, the brachial and ankle pulse waves were determined with inflatable cuffs with the pressure maintained at 30–50 mmHg to ensure that the cuff pressure had a minimal effect on the systemic hemodynamics. The blood pressure and pulse pressure were determined simultaneously [2]. The measurements were made with the patient in the supine position. The mean arterial blood pressure (MABP: mmHg) and pulse pressure (mmHg) were determined by the following formulae:

$$ \begin{array}{l}\mathrm{MABP} = \mathrm{diastolic}\ \mathrm{blood}\ \mathrm{pressure} + \left(\mathrm{systolic}\ \mathrm{blood}\ \mathrm{pressure}-\mathrm{diastolic}\ \mathrm{blood}\ \mathrm{pressure}\right)/3.\\ {}\mathrm{Pulse}\ \mathrm{pressure} = \mathrm{systolic}\ \mathrm{blood}\ \mathrm{pressure} - \mathrm{diastolic}\ \mathrm{blood}\ \mathrm{pressure}.\end{array} $$

## LSFG measurements

We calculated the parameters using LSFG Analyzer software (v.3.0.47, Softcare Co., Fukuoka, Japan). The software program then separated out the vessels using the automated definitive threshold and divided the ONH into the vessel area and the capillary area. The details of the determination of the LSFG measurements from fundus images were described previously [16, 26]. In short, LSFG uses the mean blur rate (MBR) as an indicator of the relative velocity of erythrocytes. The skew and BOT (as items of the pulse waveform analyses) were calculated. For the evaluation of the capillary area of the ONH, a circle was set surrounding the ONH. First, 118 MBR images (118 frames) were recorded from the circle and the rectangular area within a 4-s period tuned to the cardiac cycle. A gray-scale map of the still images was then made by averaging the MBR images. On the analysis screen, the pulse wave of the changing MBR values, which corresponded to each cardiac cycle, was obtained. The analysis of the screen, which is normalized to one pulse, is then displayed, and the analysis of the pulse waveform and average MBR are made on this screen.

A similar explanation of the BOT values were obtained from the waveform analysis [16, 19, 27, 28]. Skew quantifies the asymmetry of the waveform distribution, varying with the bias of the waveform shape. Specifically, the skew equals zero if the waveform is completely symmetrical, and the skew becomes positive or negative if the waveform is distributed leftward or rightward of the center point of the waveform [16].

Only the data from the right eye were used for the analysis.

## Measurements of laboratory profile

The laboratory profile of each patient was comprised of the determination of the following: glycated hemoglobin A1c (HbA1c: %), homeostasis model assessment of insulin resistance (HOMA-IR), apolipoprotein-A1 (mg/dL), apolipoprotein-B (mg/dL), hematocrit (%), platelet count (×104/μL), cystatin C (mg/L) and urinary albumin concentration (UAC: mg/L) as an indicator of kidney function, and the total cholesterol (mg/dL) obtained from fasting morning blood and urine samples. The HbA1c is expressed based on the scale of the National Glycohemoglobin Standardization Program. HOMA-IR = fasting insulin (IU/ml) × fasting blood glucose (mmol/L)/22.5 [29]. Blood and urine samples were centrifuged immediately after collection at 4,000 *g* for 10 min, and the supernatant was frozen immediately in polypropylene tubes and stored at −80 °C until its use in assays.

## Measurements of other systemic parameters and other ocular parameters

The following parameters were measured: body mass index (BMI: kg/m^2^), spherical refraction (diopters) assessed with the TONOREF 2 system (NIDEK, Aichi, Japan), ocular perfusion pressure (OPP, mmHg), and heart rate (beats per min). The OPP was defined as: (2/3MABP) – intraocular pressure. The intraocular pressure was obtained by applanation tonometry.

## Statistical analyses

Data are presented as the means ± SD for the continuous variables. Pearson’s correlation coefficients and multiple regression analysis were used to determine independent factors for skew and BOT values in the capillary area of the ONH.

We considered *p*-values <0.05 statistically significant. The StatView program ver. 5.0 (SAS, Cary, NC) was used for the statistical analyses.

## Results

The results of the characteristics of all patients are shown in Table 1. Eighty-two of the 130 (63 %) patients had hypertension, and 32 of the 130 patients (25 %) had diabetes mellitus. The mean ± SD CAVI was 8.9 ± 1.1; the mean ± SD skew was 13.1 ± 1.9, and the mean ± SD BOT was 46.7 ± 4.7. The results of the Pearson’s correlation analyses between the skew and the BOT in the capillary area of the ONH and all parameters are shown in Table 2.

**Table 1:** Characteristics of all patients (*n* = 130)

Characteristics	
Age (yrs)	60.5 ± 10.9
BMI (kg/m^2^)	25.4 ± 3.7
Hypertension (%)	82
Diabetes mellitus (%)	32
MABP (mmHg)	92.6 ± 11.8
Pulse pressure (mmHg)	55.8 ± 13.2
Ocular perfusion pressure (mmHg)	49.1 ± 8.0
Heart rate (beat per minute)	65.8 ± 9.7
HbA1c (%)	6.1 ± 0.8
HOMA-IR	1.9 ± 1.3
Apolipoprotein-A1 (mg/dL)	135.8 ± 21.5
Apolipoprotein-B (mg/dL)	92.2 ± 20.1
Hematocrit (%)	42.3 ± 4.0
Platelet counts (×10^4^/μL)	22.0 ± 5.5
eGFR (ml/min/1.73 m^2^)	69.8 ± 14.2
CAVI	8.9 ± 1.1
Range	6.3–11.8
Echo graphical characteristics	
Mean IMT (mm)	0.80 ± 0.15
Plaque score	5.2 ± 4.8
E/e’ ratio	10.7 ± 3.4
LSFG measurements	
Skew-Capillary	13.1 ± 1.9
Skew-whole of the ONH	12.3 ± 1.8
BOT-Capillary	46.7 ± 4.7
BOT-whole of the ONH	49.2 ± 4.3
Anti-hypertensive medicine	
Calcium channel blocker (%)	53.1
ACEI (%)	6.2
ARB (%)	31.5
Diuretics (%)	5.4
β-blocker/α-βblocker (%)	13.3

Data given as mean ± SD or %

*ACEI* angiotensin-converting enzyme inhibitor, *ARB* angiotensinII receptor blocker, *BMI* body mass index, *BOT* blowout time, *CAVI* cardio-ankle vascular index, *eGFR* estimated glomerular filtration rate, *HbA1c* glycated hemoglobin A1c, *HOMA-IR* homeostasis model assessment of insulin resistance, *IMT* intima-media thickness, *LSFG* laser speckle flowgraphy graphy, *MABP* mean arterial blood pressure

**Table 2:** Pearson’s correlation analysis between the CAVI and all parameters (*n* = 130)

	r	*p*
Age	0.62	<0.0001
BMI (kg/m^2^)	−0.21	0.02
MABP (mmHg)	0.08	0.38
Pulse pressure (mmHg)	0.29	0.0007
Ocular perfusion pressure (mmHg)	0.10	0.27
Heart rate (beat per minute)	−0.03	0.73
HbA1c (%)	0.24	0.007
HOMA-IR	−0.11	0.22
Apolipoprotein-A1 (mg/dL)	0.05	0.55
Apolipoprotein-B (mg/dL)	−0.10	0.28
Hematocrit (%)	−0.20	0.03
Platelet counts (×10^4^/μL)	−0.26	0.002
eGFR (ml/min/1.73 m^2^)	−0.27	0.002
Mean IMT (mm)	0.42	<0.0001
Plaque score	0.37	<0.0001
E/e’ ratio	0.21	0.02
Skew-Capillary	0.39	<0.0001
Skew-whole of the ONH	0.32	0.0002
BOT-Capillary	−0.51	<0.0001
BOT-whole of the ONH	−0.44	<0.0001

*BMI* body mass index, *BOT* blowout time, *CAVI* cardio-ankle vascular index, *eGFR* estimated glomerular filtration rate, *HbA1c* glycated hemoglobin A1c, *HOMA-IR* homeostasis model assessment of insulin resistance, *IMT* intima-media thickness, *MABP* mean arterial blood pressure

Skew was significantly correlated with age (*r* = 0.49, *p* < 0.0001), BMI (*r* = −0.23, *p* = 0.008), pulse pressure (*r* = 0.32, *p* = 0.0002), spherical refraction (*r* = 0.32, *p* = 0.0002), heart rate (*r* = −0.59, *p* < 0.0001), HbA1c (*r* = 0.21, *p* = 0.02), hematocrit (*r* = −0.28, *p* = 0.001), mean IMT (*r* = 0.36, *p* < 0.0001), the E/e’ ratio (*r* = 0.31, *p* = 0.0004) and the CAVI (*r* = 0.39, *p* < 0.0001). Cystatin C tended to be correlated with the skew but did not reach significance (*r* = 0.15, *p* = 0.09).

The BOT was significantly correlated with age (*r* = −0.64, *p* < 0.0001), BMI (*r* = 0.27, *p* = 0.002), pulse pressure (*r* = −0.38, *p* < 0.0001), spherical refraction (*r* = −0.29, *p* = 0.0008), heart rate (*r* = 0.45, *p* < 0.0001), HbA1c (*r* = −0.22, *p* = 0.01), hematocrit (*r* = 0.30, *p* = 0.0006), cystatin C (*r* = −0.19, *p* = 0.03), UAC (*r* = −0.18, *p* = 0.04), mean IMT (*r* = −0.46, *p* < 0.0001), the E/e’ ratio (*r* = −0.32, *p* = 0.0002) and the CAVI (*r* = −0.51, *p* < 0.0001).

Tables 3 and 4 shows the results of our multiple regression analysis for factors that independently contribute to the skew and BOT in the capillary area of the ONH. The factors contributing independently to the skew (*R* = 0.78, *p* < 0.0001) were heart rate (standard regression = −0.49, *t* = −8.02, *p* < 0.0001), spherical refraction (standard regression = 0.24, *t* = 3.89, *p* = 0.0002) and CAVI (standard regression = 0.18, *t* = 2.61, *p* = 0.01). The factors that were contributing independently to the BOT (*R* = 0.78, *p* < 0.0001) were heart rate (standard regression = 0.32, *t* = 5.15, *p* < 0.0001), the CAVI (standard regression = −0.27, *t* − 3.92, *p* = 0.0002), UAC (standard regression = −0.17, *t* = −2.84, *p* = 0.005), mean IMT (standard regression = −0.19, *t* = −2.74, *p* = 0.007), spherical refraction (standard regression = −0.16, *t* = −2.60, *p* = 0.01), BMI (standard regression = 0.16, *t* = 2.45, *p* = 0.02) and pulse pressure (standard regression = −0.15, *t* = −2.23, *p* = 0.0001).

**Table 3:** Results of the multiple regression analysis for factors independently contributing to the CAVI, using the blowout time in the capillary (*n* = 130)

Explanatory variable	Standard regression	*t*	*p*
BOT-Capillary	−0.28	−3.04	0.003
Platelet counts	−0.21	−2.87	0.005
eGFR	−0.16	−2.10	0.04
HbA1c	0.16	2.09	0.04
Mean IMT	0.17	2.01	0.046
Plaque score	0.11	1.34	0.18
BMI	−0.09	−1.07	0.29
Pulse pressure	0.04	0.53	0.60
Hematocrit	0.02	0.30	0.77
E/e’ ratio	−0.01	−0.15	0.88

Objective variable: CAVI. *BOT* blowout time, *CAVI* cardio-ankle vascular index, *eGFR* estimated glomerular filtration rate, *HbA1c* glycated hemoglobin A1c, *IMT* intima-media thickness, *R* = 0.64, *p* < 0.0001

**Table 4:** Results of the multiple regression analysis for factors independently contributing to the CAVI, using the skew in the capillary (*n* = 130)

Explanatory variable	Standard regression	*t*	*p*
Platelet counts	−0.22	−2.18	0.005
Mean IMT	0.21	2.54	0.01
eGFR	−0.17	−2.18	0.03
HbA1c	0.17	2.17	0.03
Skew-Capillary	0.17	2.00	0.048
Plaque score	0.14	1.65	0.10
BMI	−0.12	−1.44	0.15
Pulse pressure	0.07	0.79	0.43
Hematocrit	0.02	0.23	0.82
E/e’ ratio	−0.002	−0.03	0.98

Objective variable: CAVI, *R* = 0.63, *p* < 0.0001

*CAVI* cardio-ankle vascular index, *eGFR* estimated glomerular filtration rate, *HbA1c* glycated hemoglobin A1c, *IMT* intima-media thickness

## Discussion

This study was designed to determine whether systemic arterial stiffness shown by the CAVI significantly affects the skew and BOT, which are factors derived from a pulse waveform analysis, in the capillary area of the ONH. In our previous research, we reported that the BOT of the entire ONH was significantly correlated with age, mean IMT, left ventricular diastolic function, kidney function and the CAVI in the healthy subjects [17, 18, 30, 31]. However, in that research we examined only a single correlation to determine the relationship between the BOT and the CAVI [18].

The novel analysis method of LSFG can be used to separate the ONH into vessel and capillary areas for more detailed analyses of the ONH microcirculation. It was reported that the skew and BOT values in the capillary area of the ONH are important predictive factors for normal-tension glaucoma and silent brain infarction [19, 20].

Based on the above-mentioned findings, we hypothesized that the systemic arterial morphological and functional status also affects the skew and BOT values in the capillary area of the ONH. The present study’s Pearson’s correlation analysis and multiple regression analysis revealed that heart rate, spherical refraction and the CAVI are factors that contribute to the skew. We also observed that the skew in the capillary area of the ONH increases in parallel with the increase in the CAVI. In other words, the peak of the MBR — as an indicator of the velocity of the erythrocytes — in the cardiac cycle moves leftward in parallel with the exacerbation of the CAVI.

Our multiple regression analysis revealed that heart rate, the CAVI, UAC, mean IMT, spherical refraction, BMI and pulse pressure were factors contributing to the BOT. The BOT in the capillary area of the ONH decreased in parallel with the increase in the CAVI, UAC, mean IMT and pulse pressure. The BOT in the capillary area of the ONH was significantly correlated with these parameters, as was BOT in the entire ONH, as we reported previously [17, 18, 30, 31]. We reported that the BOT in the entire ONH is significantly negatively correlated with the absolute value of systemic vascular resistance [32]. We thus suggested that exacerbation of the CAVI, UAC and mean IMT — which indicates systemic arteriosclerosis — affects the sclerosis of the microcirculation in the capillaries of the ONH. The heart rate and CAVI in particular were revealed as factors contributing to both the skew and BOT. These results suggested that large arterial function, which is manifested in the CAVI, contributes to smooth hemodynamics of microcirculation, which is shown by the LSFG. In addition, our findings indicated that the CAVI might be a more important factor contributing to the skew and BOT in the capillary area of the ONH than the carotid arterial thickness, kidney function and left ventricular diastolic function.

Several studies have reported that the CAVI increases not only with age but also with arteriosclerotic diseases, such as coronary artery disease, carotid arteriosclerosis, chronic kidney disease, and cerebrovascular disease [33–35]. Our prior study revealed that the CAVI is an independent risk factor for exudative age-related macular degeneration, which is a retinal and choroidal vascular disease [36]. In addition, it is possible that cardiovascular disease and cerebrovascular diseases are associated with normal tension glaucoma [37, 38]. It was also reported that ischemic changes in brain magnetic resonance imaging are more common in patients with normal-tension glaucoma than in control subjects [38, 39]. It is thus clear that important risk factors for cardiovascular disease, cerebrovascular disease and normal-tension glaucoma overlap. As overlapping risk factors, we speculated in previous studies that the skew and BOT in the capillary area of the ONH are risk factors for normal-tension glaucoma and silent brain infarction [19, 20].

Our present findings also revealed that the systemic arterial function shown by the CAVI is one of the important contributors to ocular microcirculation. In other words, the microcirculation of the capillary area of the ONH calculated by LSFG provides a window for detecting the conditions of systemic large arteries. We believe our findings may be clues to the relationships between cardiovascular and cerebrovascular disease and ocular diseases, such as normal-tension glaucoma and retinal and choroidal vascular diseases.

In the present study, BMI was significantly positively correlated and spherical refraction was negatively correlated with BOT. These results provide new knowledge, and further detailed evaluations are needed to confirm these results. In any case, BMI and spherical refraction should be considered when interpreting blood flow data using LSFG. We excluded them from the multiple regression analysis, because the CAVI was strongly correlated with age (*r* > 0.60, *p* < 0.0001).

Our study has some severe limitations. First, only men were included in the study, and our findings should not be generalized to women. Second, the optic-nerve head has double blood circulation; one depends on branches of the central retinal artery while the other depends on the branches of the short posterior ciliary arteries. Both circulation routes function at different perfusion pressures [40]. We evaluated these two differences in arterial blood flow of the ONH calculated by LSFG. It is known that the arteries supplying the eye have relatively high blood pressure. The pressure drop from the small arteries and arterioles to the capillaries is greater in the choroid than in the retina [41]. At the origin of the retina and ciliary arteries, blood pressure is about 65–70 mmHg. There is a direct relationship between vessel calibre and pressure of intraocular vessels [40]. Thus, another method for evaluating these two blood flows separately could need more strict research. Third, because the design of the study was cross-sectional, we did not investigate whether the skew and BOT changed simultaneously with the changes in the CAVI. A prospective study should evaluate whether these parameters can detect changes in the CAVI. Further careful validation studies with larger patient populations of both genders are needed to evaluate whether these ocular microcirculation parameters obtained using LSFG could be used as novel markers for systemic large arterial function.

In conclusion, the CAVI was revealed as an independent factor contributing to both the skew and BOT in the capillary area of the ONH. Our study clarified that large arterial function shown by the CAVI contributes to smooth hemodynamics of microcirculation, which is shown by LSFG.
